# Factors affecting mortality and reoperations in high-energy pelvic fractures

**DOI:** 10.1007/s00590-018-2203-1

**Published:** 2018-04-19

**Authors:** Peyman Bakhshayesh, Lars Weidenhielm, Anders Enocson

**Affiliations:** Department of Molecular Medicine and Surgery (MMK), Karolinska Institute, 17176 Stockholm, Sweden

**Keywords:** Pelvic bone, Intention, Injury severity score, Glasgow coma scale, Orthopaedic surgery, Reoperation, Accidents/mortality

## Abstract

**Aim:**

Factors affecting mortality during the first year following high-energy pelvic fractures has not been reported previously. Nor has surgical complications leading to reoperations been reported in a cohort with only high-energy pelvic trauma patients.

**Objectives:**

The aim of this study was to report and analyse factors affecting outcome, in terms of mortality and reoperations, up to 1 year after the injury in patients with a traumatic pelvic ring injury due to a high-energy trauma.

**Materials and methods:**

Data from the SweTrau (Swedish National Trauma Registry) on patients admitted to the Trauma Centre Karolinska in Stockholm, Sweden, were collected. Inclusion criteria were adults (age ≥ 18), trauma with a high-energy mechanism, alive on arrival, Swedish personal identification number, reported pelvic fracture on CT scan. Patient records and radiographies were reviewed. The study period was 2011–2015 with 1-year follow-up time. Univariate and regression analysis on factors affecting mortality was performed. Risk of reoperation was analysed using univariate and case-by-case analysis.

**Results:**

We included 385 cases with mean age 47.5 ± 20.6 years (38% females): 317 pelvic fractures, 48 acetabular fractures and 20 combined injuries. Thirty-day mortality was 8% (30/385), and 1-year mortality was 9% (36/385). The main cause of death at 1 year was traumatic brain injury (14/36) followed by high age (> 70) with extensive comorbidities (8/36). Intentional fall from high altitude (OR 6, CI 2–17), GCS < 8 (OR 12, CI 5–33) and age > 70 (OR 17, CI 6–51) were factors predicting mortality. Thirty patients (22%, 30/134) were further reoperated due to hardware-related (*n* = 18) or non-hardware-related complications (*n* = 12). Hardware-related complications included: mal-placed screws (*n* = 7), mal-placed plate (*n* = 1), implant failure (*n* = 6), or mechanical irritation from the implant (*n* = 4). Non-hardware-related reasons for reoperations were: infection (*n* = 10), skin necrosis (*n* = 1), or THR due to post-traumatic osteoarthritis (*n* = 1).

**Conclusion:**

Non-survivors in our study died mainly because of traumatic brain injury or high age with extensive comorbidities. Most of the mortalities occurred early. Intentional injuries and especially intentional falls from high altitude had high mortality rate. Reoperation frequency was high, and several of the hardware-related complications could potentially have been avoided.

## Introduction

Traumatic pelvic ring injury (TPRI) is a collection name for pelvic and acetabular fractures. The incidence has been reported to be 17–37/100,000 person-years [1–5], and the high-energy TPRIs have a reported incidence of 10/100,000 person-years [1]. TPRIs have historically been considered serious injuries resulting in a mortality rate of 7–47% in poly-trauma patients [1, 6–11]. Pelvic bleeding has been reported as a major contributor to mortality in these patients [11]. However, during the last decades several authors have found that concomitant injuries such as head or chest injury, with or without bleeding, can significantly affect the mortality rather than solely bleeding caused by the pelvic fracture [12, 13]. Not all of these injuries are caused by non-intentional accidents, but some are actually caused by intentional acts by the patients, a fact that is rarely discussed or analysed in the existing literature. Most of the deaths in poly-trauma patients happen early during the first 30 days, and therefore this parameter has been commonly used in trauma research. Later follow-ups are more difficult to perform, and mortality between 30 days and 1 year has not been reported as an outcome in high-energy poly-trauma patients previously. In the survivor group, an appropriate definitive fixation is of value and reoperation might prolong rehabilitation and return to normal daily activity [14]. Definitive fixation of TPRIs consists of restoration of the pelvic anatomy by means of plates, screws, rods, or external fixators [15–17]. There are several published articles addressing implant-related complications following a single method, such as sacro-iliac (SI) screws, posterior tension band plating, posterior iliolumbar fixation [18–21]. However, there are only a few reports on overall risks for reoperations without in detail description following pelvic or acetabular fracture surgery in an unselected population of high-energy trauma patients [22, 23].

### Aim

The aim of this study was to report and analyse factors affecting outcome, in terms of mortality and reoperations, up to 1 year after the injury in patients with a TPRI due to a high-energy trauma.

## Materials and methods

The trauma unit at the Karolinska University Hospital is the primary receiving unit for all high-energy trauma victims in the Stockholm region with a catchment population of around 2.3 million [24]. All patients with a high-energy TPRI admitted as trauma alerts to the Karolinska University Hospital during 2011–2015 were identified via the SweTrau registry. The SweTrau is a Swedish national trauma registry with a coverage of almost 70% of all poly-trauma patients in the country and > 80% in the Stockholm’s county [25]. Criteria for registration in the SweTrau registry are trauma victims accepted to the trauma units when a trauma alert has been triggered [25]. A TPRI was defined as a bony or ligamentous injury to the pelvic ring (sacrum, coccyx, ischium, pubic bone, or innominate bones including acetabulum). Patient records were searched until 31 December 2016 or death, giving a minimum follow-up time of 1 year.

Initially, a total of 8453 trauma records for the period 2011–2015 were identified. Each trauma case had up to 10 diagnoses based on the ICD 10 (International Classification of Disease) in their records. Patients with diagnoses including S32 and S33 were selected, resulting in 750 patients. Spinal fractures with ICD code S32.00 but without a TPRI were subsequently excluded. This left us with 586 patients whose records were reviewed with respect to the following inclusion criteria:

1. High-energy injury mechanism, defined as road and traffic-related injuries, high falls, industrial and agricultural injuries [1].
2. Injury to the pelvic ring on CT scan.
3. Patients alive on arrival (based on existing vital signs).
4. Swedish personal ID-number.
5. Age ≥ 18 years on admission.

Non-survivors were compared with survivors (control group). (Patients’ characteristics are presented in Table 1a.) Surgically treated patients were divided into those who underwent a reoperation and those who did not (control group). (Patients’ characteristics are presented in Table 1b.) A reoperation was defined as an unexpected event following primary surgery demanding new surgery during the study period. Cause of death was analysed based on review of the patient records and the death report. High age was defined as age > 70. Extensive comorbidities were defined as patients with one or more systematic disease. Fracture patterns were analysed and classified using the initial trauma CT scan. Pelvic fractures were classified using the Young–Burgess classification system [26]. Acetabular fractures were classified according to Letournel [27].

**Table 1:** Type of pelvic fracture in relation to treatment

*(a) Patients with pelvic ring injuries (Young-Burgess classification) and type of treatment* ^a^						
Fracture type	*n* = (%)	Type of treatment *n*				
		Non-surgical	Plating	SI-screw	Ex-fix	Combined
All	317 (100)	239	18	8	4	48
APC 1	5 (1.6)	4	1	0	0	0
APC 2	21 (6.6)	3	7	0	2	9
APC 3	10 (3.2)	0	1	1	0	8
LC 1	183 (58)	180	2	0	0	1
LC 2	13 (4.1)	7	2	3	1	0
LC 3	17 (5.4)	4	2	1	1	9
Vertical shear	62 (20)	39	2	3	0	18
Combined	6 (1.9)	2	1	0	0	3

*(b) Patients with acetabular fractures (Letournel classification) and type of treatment* ^b^				
Fracture type	*n*= (%)	Type of treatment *n*		
		Non-surgical	Plating	Combined
All	48 (100)	10	33	5
Post wall	23 (48)	4	16	3
Post column	1 (2.1)	1	0	0
Ant column	1 (2.1)	0	0	1
Transverse	2 (4.2)	1	0	1
T-type	4 (8.3)	3	1	0
Transverse and post wall	5 (10)	0	5	0
Ant column post hemi-transverse	0 (0)	0	0	0
Both column	12 (27)	1	11	0

*(c) Patients with combined fractures (pelvic and acetabular) and type of treatment*				
Fracture type	*n*	Type of treatment *n*		
		Non-surgical	Plating	Combined
Combined mechanism	20	2	12	6

^a^*APC* Antero–posterior compression, *LC* Lateral compression, *Ex-fix* External fixation

^b^*Post* Posterior, *Ant* Anterior

### Statistics

The Mann–Whitney U test was used for scale variables in independent groups. Normality was tested with the Shapiro–Wilk test. Nominal variables were tested by the Chi-square test or the Fisher’s exact test. All tests were two-sided. Multivariate logistic regression analysis was performed and the results are presented as odds ratio (OR) with confidence interval (CI). The results were considered significant at *p* < 0.05. Statistical analysis was performed using IBM SPSS Statistics version 24 for Windows.

## Results

A total of 385 patients (145 females) with mean age (± SD) 47.5 ± 20.6 years fulfilled all the inclusion criteria and were included in the study. The median follow-up time was 40 (IQR 26–56) months. A majority of the patients (*n* = 251, 65%) were treated non-surgically. For those patients treated surgically, the median time to primary surgery was 3 (IQR 2–5) days.

Fracture types were: pelvic (*n* = 317), acetabular (*n* = 48), or combined (*n* = 20) (Table 1a–c). Mean age (± SD) was 48.0 (± 21.1) years for patients with pelvic fractures, 46.1 (± 18.2) years for patients with acetabular fractures, and 44.5 (± 16.7) years for patients with combined fractures. There was a greater proportion of females among patients with pelvic fractures (131/317, 41%) compared to patients with acetabular fractures (9/48, 19%) (*p* = 0.002), and 38 out of 48 (79%) acetabular fractures were operated in comparison with 78 out of 317 (25%) pelvic fractures (*p* < 0.001).

### Mortality

The overall 30-day mortality was 30/385 (7.8%) and the 1-year mortality was 36/385 (9.4%) (Table 2). A fall from high altitude was the injury mechanism in 19 out of 30 cases of 30-day mortality, and in 24 out of 36 cases of 1-year mortality. Mortality in the first 30 days was caused by: traumatic brain injury (*n* = 13), high age (> 70 years) with extensive comorbidities (*n* = 6), traumatic chest injury (*n* = 4), pelvic bleeding (*n* = 4), abdominal bleeding (*n* = 2), or chest bleeding (*n* = 1). All cases of bleeding leading to death died during the first 24 h. The cause of death for those six patients who died later than 30 days during the first year were: sepsis with multi-organ failure (*n* = 3), high age (> 70 years) with extensive comorbidities (*n* = 2), or late sequelae from a traumatic brain injury (*n* = 1). Surgically treated patients had lower 30-day mortality (2/134, 1.5%) compared to non-surgically treated patients (28/251, 11%) (*p* < 0.001). As was the 1-year mortality lower among surgically treated (4/134, 3.0%) compared to non-surgically treated patients (32/251, 13%) (*p* = 0.001).

**Table 2:** Comparison between survivors and non-survivors

		All patients *n* = 385	Survivors 1-year *n* = 355	Non-survivors *n* = 36	*p* value
Age	Mean ± SD	47 ± 21	46 ± 19	63 ± 26	< 0.001
*Gender*					
Female	*n* (%)	145 (38)	129 (37)	16 (44)	0.37
Male	*n* (%)	240 (62)	220 (63)	20 (56)	
*Fracture type*					
Pelvic	*n* (%)	317 (83)	286 (82)	31 (86)	0.939
Acetabular	*n* (%)	48 (12)	44 (13)	4 (11)	
Combined	*n* (%)	20 (5)	19 (5)	1 (3)	
*Mechanism of injury*					
MVA	*n* (%)	194 (50)	182 (52)	12 (33)	0.001
Fall	*n* (%)	154 (40)	130 (37)	24 (67)	
Others	*n* (%)	37 (10)	37 (11)	0 (0)	
*Cause of injury*					
Intentional	*n* (%)	92 (24)	78 (22)	14 (40)	0.035
Non-intentional	*n* (%)	292 (76)	271 (78)	21 (60)	
ISS	Median (IQR)	22 (14–38)	22 (13–35)	33 (17–50)	0.011
GCS	Median (IQR)	13 (13–15)	13 (13–15)	8 (3–14)	< 0.001
Systolic BP	Mean ± SD	121 ± 28	121 ± 28	114 ± 31	0.219
Respiratory rate	Mean ± SD	21 ± 8	20 ± 7	26 ± 10	< 0.001
Pulse rate	Mean ± SD	90 ± 19	90 ± 19	91 ± 18	0.895
Head & neck injury	*n* (%)	118 (31)	99 (28)	19 (54)	0.003
Chest injury	*n* (%)	168 (44)	150 (43)	18 (50)	0.481
Abdominal injury	*n* (%)	74 (19)	68 (19)	6 (17)	0.826

*MVA* All type of motor vehicle accidents, *ISS* Injury Severity Score, *GCS* Glasgow Coma Scale Score, *BP* Blood Pressure, *H&N Injury* Head and Neck Injury

### Intentional versus non-intentional cause of injury

There was an increased 30-day mortality among patients with intentional (14/92, 15%) compared to non-intentional (16/293, 5.5%) cause of injury (*p* = 0.006). There was also an increased 1-year mortality among patients with intentional (14/92, 15%) compared to non-intentional (22/293, 7.5%) cause of injury (*p* = 0.04), as no further fatalities happened in intentional group between 30 days and 1 year. Intentional trauma patients displayed higher ISS (median, IQR) (34, 22–43) compared to non-intentional patients (20, 12–33) (*p* < 0.001), and they had a greater proportion of fall from high altitude (76/92, 83%) compared to non-intentional patients (78/293, 27%) (*p* < 0.001) (Table 3). Regression analysis including factors affecting mortality was conducted. Based on 36 cases of fatalities up to 1 year, three factors were selected to predict a mortality model. We found that mechanism of injury and injury reason were confounding each other. A model based on intentional versus non-intentional (OR 4, CI 1.5–11.5), Glasgow Coma Scale (GCS) ≤ 8 (OR 11, CI 4–30), age ≥ 70 (OR 21, CI 7–61) showed an appropriate goodness of fit using Hosmer–Lemeshow test (*p* = 1.0). Nagelkerke *R*^2^ was 0.4. Another model using Mechanism of injury showed that fall from high altitude (fall) compared to motor vehicle accident (MVA) (OR 3.5, CI 1.4–9), GCS ≤ 8 (OR 14, CI 5–37), and age ≥ 70 (OR 13, CI 5–33) had reliable goodness of fit with Hosmer–Lemeshow test (*p* = 0.9) and comparable Nagelkerke *R*^2^, 0.4. A combination of Injury reason and mechanism of injury in a regression model was thus more appropriate. In this final model, intentional fall compared to non-intentional MVA had OR 6 (CI 2–17), GCS ≤ 8 OR 12 (CI 5–33), and age ≥ 70 OR 17 (CI 6–51). Hosmer–Lemshow goodness of fit test had a *p* value = 0.9 and Nagelkerke *R*^2^ was 0.4. ISS as a scale variable (*p* = 0.3) or ISS > 16 (*p* = 0.9) or ISS > 25 (*p* = 0.5) did not remain significant in a multivariate analysis.

**Table 3:** Comparison between patients with Intentional and Non-intentional injuries

		All	Intentional	Non-Intentional	*p* value
Age	Mean ± SD	47 ± 21	40 ± 16	50 ± 21	< 0.001
Female	*n* (%)	145 (38)	38 (41)	107 (37)	0.421
Pelvic	*n* (%)	316 (82)	79 (86)	237 (81)	0.067
Acetabulum		48 (13)	6 (6)	42 (14)	
Both		20 (5)	7 (8)	13 (5)	
Mechanism	*n* (%)				
MVA		194 (50)	15 (16)	179 (61)	< 0.001
Fall		153 (40)	76 (83)	77 (26)	
Others		37 (7)	1 (0)	36 (10)	
ISS	Median (IQR)	22 (14–38)	34 (22–43)	20 (14–38)	< 0.001
GCS	Median (IQR)	15 (13–15)	14 (8–15)	15 (14–15)	< 0.001
H&N injury	*n* (%)	118 (29)	32 (35)	86 (29)	0.302
Chest injury	*n* (%)	168 (44)	50 (54)	118 (40)	0.022
Abdominal injury	*n* (%)	74 (19)	24 (26)	50 (17)	0.069
HLOS	Median (IQR)	10 (5–23)	16 (10–36)	9 (5–20)	< 0.001
ICULOS	Median (IQR)	1 (0–5)	2 (0–9)	0 (0–4)	< 0.001
ORIF	*n* (%)	134 (35)	36 (39)	98 (34)	0.38
30-day mortality	*n* (%)	30 (8)	14 (15)	16 (5.5)	0.006
1-year mortality	*n* (%)	35 (9)	14 (15)	21 (7)	0.035
Time to death	Median (IQR)	4.5 (1–15)	1.5 (1–9)	5 (1–26)	0.18

*MVA* All type of motor vehicle accidents, *ISS* Injury Severity Score, *GCS* Glasgow Coma Score, *H&N Injury* Head and Neck Injury, *HOLS* Hospital Length of Stay, *ICULOS* Intensive Care Unit Length of Stay, *ORIF* Open Reduction and Internal Fixation

### Reoperations

Thirty out of 134 (22%) surgically treated patients were reoperated (Table 4). The reasons for reoperations were hardware-related complications in 18 patients and non-hardware-related complications in 12 patients. Hardware-related complications included: mal-placed screws (*n* = 7), mal-placed plate (*n* = 1), implant failure (*n* = 6), or mechanical irritation from the implant (*n* = 4). Non-hardware-related reasons for reoperations were: infection (*n* = 10), skin necrosis (*n* = 1), or total hip replacement (THR) due to post-traumatic osteoarthritis despite an adequate fixation (*n* = 1).

**Table 4:** Comparison of characteristic factors in reoperated and non-reoperated patients

		All surgically treated *n* = 134 (100%)	No reoperation *n* = 104 (78%)	Reoperation *n* = 30 (22%)	*p* value
Age	Mean ± SD	45 ± 16	45 ± 16	45 ± 15	0.875
*Gender*					
Female	*n* (%)	37 (28)	29 (28)	8 (27)	1.0
Male	*n* (%)	97 (72)	75 (72)	22 (73)	
*Fracture type*					
Pelvic	*n* (%)	78 (58)	58 (56)	20 (67)	0.635
Acetabular	*n* (%)	38 (28)	31 (30)	7 (23)	
Combined	*n* (%)	18 (14)	15 (14)	3 (10)	
*Mechanism of injury*					
MVA	*n* (%)	77 (58)	60 (58)	17 (56)	0.14
Fall	*n* (%)	46 (34)	38 (36)	8 (27)	
Others	*n* (%)	11 (8)	6 (6)	5 (17)	
*Cause of injury*					
Intentional	*n* (%)	36 (27)	29 (28)	7 (23)	0.815
Non-intentional	*n* (%)	98 (73)	75 (72)	23 (77)	
ISS	Median (IQR)	29 (17–42)	29 (17–41)	30 (19–42)	0.837
GCS	Median (IQR)	15 (13–15)	15 (13–15)	15 (14–15)	0.407
Systolic BP	Mean ± SD	118 ± 30	120 ± 30	115 ± 29	0.536
Respiratory rate	Mean ± SD	21 ± 8	21 ± 7	23 ± 11	0.406
Heart rate	Mean ± SD	92 ± 19	92 ± 20	91 ± 19	0.783
H&N injury	*n* (%)	28 (21)	24 (23)	4 (13)	0.314
Chest injury	*n* (%)	63 (47)	50 (48)	13 (43)	0.683
Abdominal injury	*n* (%)	34 (25)	26 (25)	8 (27)	0.817
Time to first surgery	Median (IQR)	3 (2–5)	3 (2–5)	3 (2–5)	0.629
BMI	Median (IQR)	25 (22–29)	25 (22–29)	24 (22–31)	0.846
HLOS	Median (IQR)	18 (10–27)	18 (10–25)	23 (10–44)	0.282
ICULOS	Median (IQR)	2 (0–10)	2 (0–10)	1 (1–14)	0.485
Mortality 30 day	*n* (%)	2 (1.5)	2 (2)	0 (0)	1.0
Mortality 1 year	*n* (%)	4 (3)	3 (3)	1 (3)	1.0

*MVA* All type of motor vehicle accidents, *ISS* Injury Severity Score, *GCS* Glasgow Coma Score, *H&N Injury* Head and Neck Injury, *HOLS* Hospital Length of Stay, *ICULOS* Intensive Care Unit Length of Stay, *ORIF* Open Reduction and Internal Fixation

**Fig. 1:**
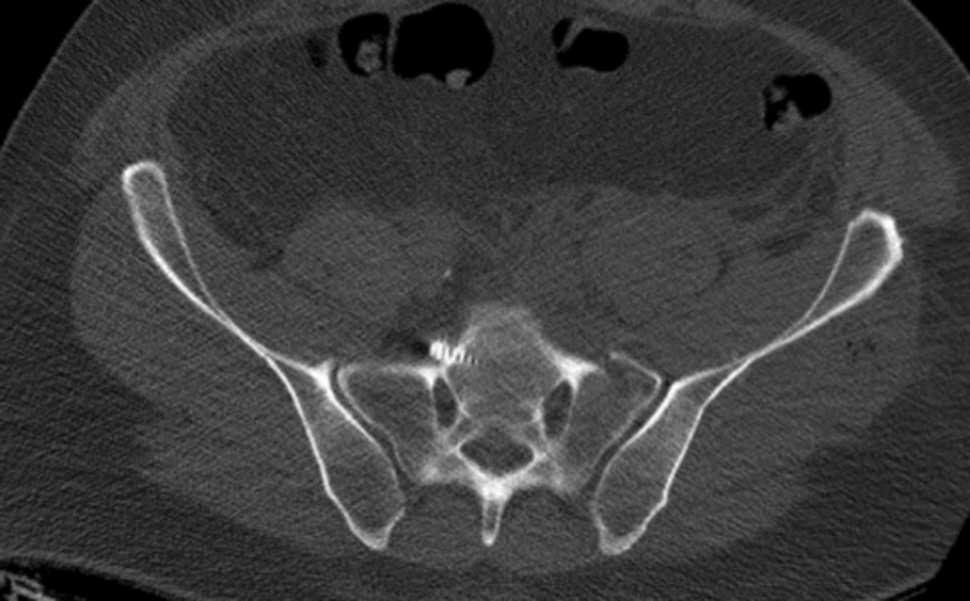
Anterior penetration of contralateral anterior sacral wall with L5-root palsy

**Fig. 2:**
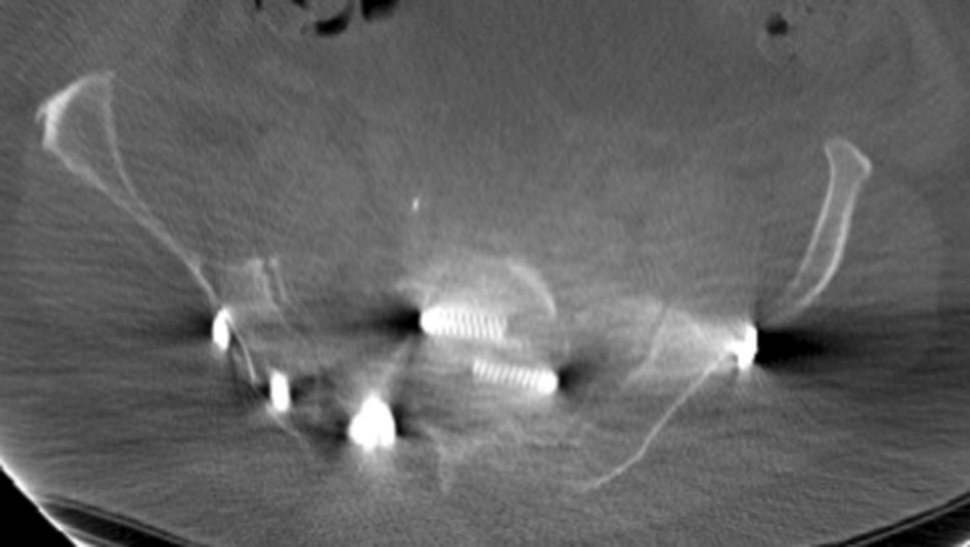
Penetration of posterior SI-screw in the S1-canal

**Fig. 3:**
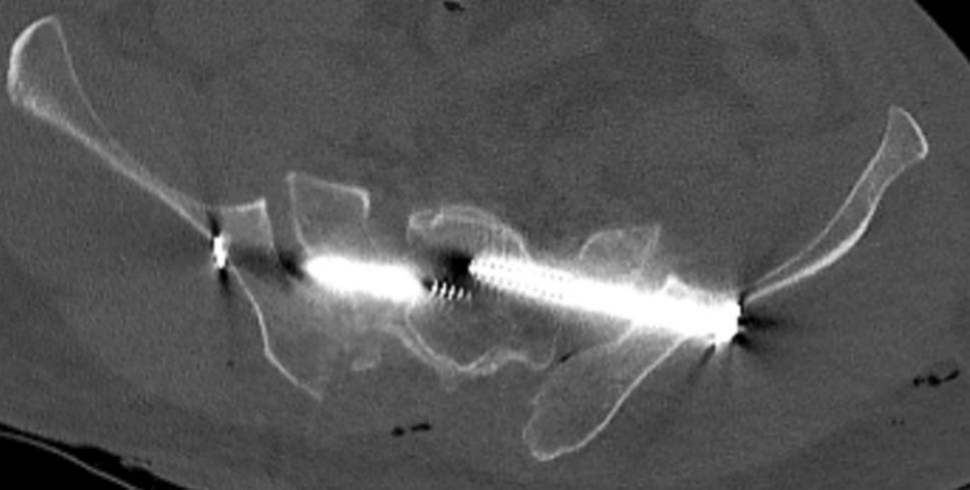
Anterior sacral dislocation and insertion of bilateral SI-screws causing nerve palsies

The eight patients with mal-placed implants consisted of three patients with SI-screws, of which 1 SI-screw penetrated to the ipsilateral S1 root, 1 SI-screw penetrated the contralateral sacral wall inducing L5-root symptom and the third case was bilateral SI-screws causing anterior displacement of the sacral body causing L5-S1 symptoms (Figs. 1, 2, 3). In addition, there were three cases of screw penetration to the hip joint following anterior column fixation of acetabular fractures, whereof 2 were found early on the post-operative CT scans, and 1 was found during THR surgery 2.5 years later. Finally, there was 1 case with 2 SI-screws who needed screw tightening. The last case of mal-placed implant was an anterior SI-joint plate placed too medial and thereby causing local L5-root symptom. Implant failure occurred in six cases: 3 posterior wall acetabular fractures with loss of reduction, 2 anterior symphyseal plates with loss of reduction and 1 plated trans iliac wing fracture with loss of reduction. Details on patients reoperated due to hardware-related problems are given in Table 5. The median (IQR) hospital length of stay was 23 (10–44) days for patients who underwent a reoperation during their primary hospital stay, compared to 18 (10–25) for surgically treated patients who were not reoperated (*p* = 0.2).

**Table 5:** Hardware related complications in 18 patients

No	Gender	Age	Fracture type	Type of primary surgery	Indication for reoperation	Time to reoperation	Type of reoperation
1	Male	67	Acetabulum Transverse-post wall	Post wall plate	Implant failure Loss of reduction and hip dislocation	23 days	Replating
2	Male	45	Acetabulum Post wall	Post wall plate	Implant failure Hip dislocation and signs of OA	47 days	THR
3	Male	20	Pelvic LC 3	SI-screw and iliac wing plate	Implant failure Fracture dislocation of right iliac wing (Insufficient fixation)	19 days	Replating
4	Male	46	Acetabulum Post wall	Post wall plate	Implant failure Loss of reduction	5 days	Replating
5	Male	59	Pelvic LC 2	Anterior symphyseal plate	Implant failure Anteriorly and loss of reduction	46 days	Screw removal
6	Female	49	Pelvic LC 3	SI-screw and symphyseal plate	Implant failure Anteriorly and loss of reduction	15 days	Replating
7	Male	28	Pelvic LC 3	SI-screws (S1 and S2)	Mechanical irritation Locally related to SI-screws	2 years	SI-screws removal
8	Male	61	VS fracture and symphyseal widening	Iliolumbar instrumentation	Mechanical irritation Loss of reduction and prominent iliac screws	3 years	Post tension band removal
9	Male	54	CM (Bilateral acetabulum, post wall), VS pelvic fracture	Iliolumbar instrumentation and post wall acetabular plates	Mechanical irritation Prominent iliac screws	1.5 years	Post tension band removal
10	Male	46	Pelvic LC 3	2 anterior SI-plates, 1 iliac wing plate, 1 anterior lag screw	Mechanical irritation Single lag screw	3 years	Lag screw removal
11	Male	49	CM	Bilateral SI-screws, Iliolumbar instrumentation and anterior plate	Mal-placed SI-screw Lt SI-screw disturbed S1-root	6 days	SI-screw, reinsertion
12	Male	51	Pelvic APC 3	Bilateral SI-screws and anterior lag screws	Mal-placed SI-screw Lt SI-screw disturbed L5-S1 roots	8 days	SI-screw changed to ant SI-plates
13	Male	46	Pelvic LC 2	SI-screw	Mal-placed SI-screw Anterior sacral wall penetration and L-5 root symptom	2 days	SI-screw reinsertion
14	Female	46	Pelvic LC 3	Iliac wing plate, symphyseal plate and, 2 SI-screws	Mal-placed SI-screw SI-screws needed further tightening	2 days	SI-screw reinsertion
15	Female	62	Pelvic LC 3	SI-screw, SI-plates and symphyseal plate	Mal-placed SI-plate Irritating L5-roots	6 month	SI-plates removal and SI-screw insertion
16	Male	23	CM including double column acetabular fracture	Iliac wing plate and post wall plate	Screw penetration to acetabulum	6 days	Reinsertion of mal-placed screw
17	Male	28	Acetabulum Both column	Iliac wing plates and separate post column screws	Screw penetration to acetabulum	3 days	Reinsertion of mal-placed screw
18	Male	26	CM, including double column acetabular fracture	Iliac wing plates and separate lag screws	Screw penetration to acetabulum	2.5 years	THR

*LC* Lateral Compression (Young–Burgess classification), *APC* Antero-Posterior Compression (Young–Burgess classification), *CM* Combined Mechanism, *THR* Total Hip Replacement

## Discussion

The main finding of this study was that the overall mortality was relatively low, but for a subgroup of patients, those with an intentional trauma mechanism and fall from high altitude, the mortality was severely elevated. Regression analysis revealed that intentional injuries (particularly intentional falls) and GCS < 8 and high age (> 70) were predictors of mortality. Traumatic brain injury was the main cause of mortality. The reoperation rate was considerable, and several of the reoperations could possibly have been avoided.

### Epidemiology

The incidence of pelvic fractures in our study was comparably lower, estimated to 3.6/100,000 person-years, than the previously reported incidence of high-energy pelvic trauma of 10/100,000 person-years, and this may be due to a lower incidence of motor vehicle accidents [1]. Pelvic fractures were more common than acetabular fractures in our material, and this is in agreement with other reports [8, 28]. Pelvic fractures were proportionally more common among women, a finding that has been reported by others [1, 3]. Fall injuries were more common (40%) compared to previous published data (5–36%) [1, 9, 16, 29]. However, our results were comparable to epidemiological findings in Finland by Lüthje et al. [3] who reported 51% fall injuries. The high proportion of fall in our cohort can be explained by the fact that other causes, such as traffic accidents, were less common in our material compared to other reports [1, 29, 30].

### Mortality

Our overall 30-day mortality rate was low (8%) compared to most other reports (7–47%) [1, 6–8, 10, 11]. Furthermore, the mortality rate increased with only 1% between 30 days and 1 year. To our knowledge, this has not been reported previously. The most common cause of death was not bleeding associated with the pelvic injury, but a traumatic brain injury. Our results might reflect the fact that substantial numbers of our cohort were intentionally injured patients (24%) and a fall from high altitude was a common trauma mechanism (40%). Sweden is relatively safe country with respect to traffic injuries, and the fact that head injuries or GCS < 8, age > 70 and intentional falls were predictors of mortality in our study might only give information about our population of study, and our results should primarily be compared with data from similar trauma populations.

### Intentional versus non-intentional cause of injury

We found an increased rate of 30-day, as well as 1-year, mortality for those with an intentional trauma mechanism compared to for the non-intentional cases. This could be due to several reasons. Deaths related to traumatic head injuries among cases with intentional injuries in our material could be related to more cases landing on their heads compared to Gabbe et al. [31], while their higher mortality rate (48%) could be explained by more severely injured cases as they included only cases with ISS > 16, while we included cases based on the mechanism of injury (high-energy trauma). However, we were not able to show any effect of ISS as a predictor of mortality in our study.

### Reoperations

One hundred and thirty-five of the 385 patients were operated, and of those 30 patients were reoperated. The reasons were mainly hardware-related complications or infections. Unfortunately, there are only few reports regarding overall reoperation frequency of high-energy TPRI patients. We consider our overall reoperation rate as high, but it is in level with other reports [14, 22]. Several of our implant complications consisted of mal-placed implants (*n* = 8) or implant failures (*n* = 6). With better preoperative planning and better intra-operative imaging techniques, we think that the majority of these reoperations might have been avoidable. In our cohort 24 cases with “posterior wall” and “transverse and posterior wall” were operated. Of those, we had three cases of implant failure. All suffered from multi-fragmentation of the posterior wall, a fact that previously been highlighted by Saterbak et al. [21] who in a retrospective study of 42 cases with posterior wall acetabular fractures reported 26% implant failure with loss of reduction. In their series multi-fragmentation of the posterior wall and fractures into the subchondral arc was reported as predictors for reoperation. Two cases of 22 with anterior symphyseal plate in our series underwent reoperation because of failure. Morris et al. [32] in a retrospective study of 148 cases with anterior symphyseal plates reported 42% implant failure, but the majority of these failures were asymptomatic.

### Strengths and limitations

One strength of our study was that no case was lost to follow-up. This was because of linkage between Swedish Population Registry and the hospital’s patient record system which made it possible to report 1-year mortality. Another strength of our study is the unique Swedish personal identification number which enabled us to follow all the cases in different data systems such as patient records, radiology data system. Our study had some clear limitations. Its retrospective design and lack of pre-designed control groups were some of our limitations. Another weakness of our study was the heterogeneous trauma panorama of the patients.

## Conclusion

Non-survivors in our study died mainly because of traumatic brain injury, or high age with extensive comorbidities. Most of the mortalities occurred early. Intentional injuries and especially intentional falls had high mortality rate in our study. Reoperation frequency was high but in the level with previous reports. A majority of the hardware-related complications could potentially have been avoided.
